# The synthesis and coupling of photoreactive collagen-based peptides to restore integrin reactivity to an inert substrate, chemically-crosslinked collagen

**DOI:** 10.1016/j.biomaterials.2016.01.044

**Published:** 2016-04

**Authors:** Jean-Daniel Malcor, Daniel Bax, Samir W. Hamaia, Natalia Davidenko, Serena M. Best, Ruth E. Cameron, Richard W. Farndale, Dominique Bihan

**Affiliations:** a Department of Biochemistry, University of Cambridge, Downing Site, Cambridge, CB2 1QW, UK; b Department of Materials Science and Metallurgy, University of Cambridge, 27 Charles Babbage Road, Cambridge, CB3 0FS, UK

**Keywords:** Biomimetic material, Photoreactive triple-helical peptide, Cell adhesion, Cell spreading, HT1080, Rugli

## Abstract

Collagen is frequently advocated as a scaffold for use in regenerative medicine. Increasing the mechanical stability of a collagen scaffold is widely achieved by cross-linking using 1-ethyl-3-(3-dimethylaminopropyl)carbodiimide (EDC) and N-hydroxysuccinimide (NHS). However, this treatment consumes the carboxylate-containing amino acid sidechains that are crucial for recognition by the cell-surface integrins, abolishing cell adhesion. Here, we restore cell reactivity to a cross-linked type I collagen film by covalently linking synthetic triple-helical peptides (THPs), mimicking the structure of collagen. These THPs are ligands containing an active cell-recognition motif, GFOGER, a high-affinity binding site for the collagen-binding integrins. We end-stapled peptide strands containing GFOGER by coupling a short diglutamate-containing peptide to their N-terminus, improving the thermal stability of the resulting THP. A photoreactive Diazirine group was grafted onto the end-stapled THP to allow covalent linkage to the collagen film upon UV activation. Such GFOGER-derivatized collagen films showed restored affinity for the ligand-binding I domain of integrin α_2_β_1_, and increased integrin-dependent cell attachment and spreading of HT1080 and Rugli cell lines, expressing integrins α_2_β_1_ and α_1_β_1_, respectively. The method we describe has wide application, beyond collagen films or scaffolds, since the photoreactive diazirine will react with many organic carbon skeletons.

## 1 Introduction

Collagen is the most abundant protein in the human body and provides a structural and biological support for tissues, where cells can proliferate and differentiate. Fibrillar collagen I is a major constituent of the extracellular matrix (ECM), and is therefore an obvious choice as a scaffold material for regenerative medicine [1], [2], [3]. Its physical properties have evolved to provide the necessary strength, stiffness, and three-dimensional architecture [4], [5] to support a complex vertebrate organism. In addition, it must offer cellular recognition motifs to allow diverse cell types to attach to and maintain the ECM, as well as to fulfil their own tissue-specific activities [6], [7]. In nature, cells regulate and organize their ECM, so that collagen fibres are stabilized by intrinsic cross-links and by interacting with several polymeric proteins that also contribute to tissue architecture and stability. In tissue engineering, however, it is difficult to mimic the cohesion of the native ECM. The necessary physical integrity may be introduced to the scaffold by chemical cross-linking using 1-ethyl-3-(3-dimethylaminopropyl)carbodiimide (EDC) with N-hydroxysuccinimide (NHS) [8], [9]. Carbodiimide-mediated collagen cross-linking involves condensation between amino acid sidechains containing carboxylate (Asp, Glu) and amino (Lys, hydroxylysine) groups, which can result in a dramatic loss of cell adhesion [10], [11]. Glu especially is necessary for recognition of major collagen receptors, such as integrins α_1_β_1_ and α_2_β_1_ [12].

The functionalization of collagen scaffolds with ligands that support cellular attachment via other classes of integrin, especially the well-established Arg-Gly-Asp tripeptide (RGD), has been extensively studied [13], [14], [15]. However, the use of triple-helical peptides (THPs) mimicking the native collagen structure remains rare in tissue engineering as a consequence of their greater complexity. Although THPs promote cell attachment and growth on various surfaces [16], [17], their effect on cell activity has not, to our knowledge, been studied on natural collagen matrices. Recent studies in this lab have focused on characterizing the binding sites for collagen receptors using libraries (named Toolkits) of such synthetic THPs, homotrimeric peptides with each strand containing an active “guest” sequence flanked with five GPP “host” triplets, to drive triple helix formation [18]. Screening of Toolkits against collagen-binding integrins led to the identification of the generic sequence GxxʹGExʺ as a ligand for α_1_β_1_ and α_2_β_1_ [12], [19], [20]. Amongst those motifs, GFOGER has attracted particular interest as the major integrin binding site located in collagen I [12]. In this study, we used a model GFOGER-containing THP to provide proof-of-concept for decorating collagen matrices to restore the natural affinity of collagen for integrins α_1_β_1_ and α_2_β_1_. In contrast, RGD-containing peptides support the binding of other integrins such as α_IIb_β_3_, α_v_β_3_ and α_5_β_1_.

Here, we have optimized the synthesis of these long peptides (36-mers), and have increased the stability of the triple helix. This is achieved by establishing covalent linkage between the three peptide strands [21], [22], which has often been attempted in collagen-mimetic peptides as a means of specifying the one-residue stagger that occurs, for example, between the α_1_ and α_2_ chains of the heterotrimeric collagens I and IV [23]. Directed activation of cysteine residues to generate specific disulfide knots at the C-terminal end of the THP produced poor yields [24], while solid-phase synthesis of homotrimeric peptides elongated from α- and ε-amino groups of di-lysine [25] was hindered, in our hands, by the aggregation of the growing strands. We therefore synthesized covalently end-stapled triple helical peptides using a more attractive method recently described by Khew et al., in which the three strands are linked together whilst still on the resin support, by the three carboxylic acid groups of the fully natural hexapeptide, GFGEEG [26], [27].

We have observed passive adsorption of THPs by collagen fibres [28]. However, it is advantageous for regenerative medicine that ligands be covalently coupled to avoid elution from the collagen matrix. Although various matrices have been derivatized with THPs [29], we sought to develop an efficient and versatile method for grafting THPs to collagen-based scaffolds whilst incorporating as little unnatural or surplus chemistry as possible. The strategy we selected is to add a photo-reactive group, Diazirine, to the free N-terminus of the end-stapled peptide ligand, which can readily be activated by low energy (365 nm) UV radiation [30], [31], [32] once adsorbed onto the collagen surface. This experimental design combines high triple helix stability with a single reactive group per THP, allowing controlled decoration of the collagen film surface.

In tissue engineering, three-dimensional scaffolds are often employed, and their construction using an ice-templating freeze-drying process has been described [33], [34], [35]. Other methods for scaffold construction, not restricted to collagen, include electrospinning [36], phase separation [37], self-assembling hydrogels [38] or cell encapsulating microgels [39], reviewed by Lu et al. [40]. However, to reduce the complexity of the study, we applied our photo-reactive THP technology to two-dimensional collagen films, with a view to applying the methodology to the more complex three-dimensional collagen scaffolds in the future.

The aim of this work is therefore to produce a modified collagen I-based film, that possesses suitable structural properties through EDC/NHS cross-linking, whilst providing cell support by covalently-coupled photo-reactive synthetic THPs. We first verified that THPs retain their affinity for the collagen-binding integrins once coupled to films. We then compared cell binding and spreading on untreated films and those functionalized with covalently linked THPs.

## 2 Material and methods

### 2.1 General procedure for peptide synthesis

9-Fluorenylmethoxycarbonyl (Fmoc) protected amino acids were supplied from Merck (Darmstadt, Germany). All other amino acids and reagents were purchased from Sigma–Aldrich (Gillingham, UK) and AGTC Bioproducts (Hessle, UK). Peptides were synthesized using Fmoc/*tert*-butyl solid phase strategy on a Liberty™ microwave peptide synthesizer (CEM) on a 0.1 mmol scale. C-terminus amidated peptides were synthesized on Fmoc-Rink amide aminomethyl Tantagel resin (0.526 g, loading 0.19 mmol g^−1^, RAPP Polymere). C-terminus carboxylated peptides were synthesized on Fmoc-Gly-Wang (0.127 g, 0.79 mmol g^−1^, Novabiochem). C-terminus biotinylated peptides were synthesized on Fmoc-PEG-Biotin NovaTag resin (0.208 g, 0.48 mmol g^−1^, Novabiochem). Resins were swollen in *N,N*-Dimethylformamide (DMF) for 90 min before use. Stepwise assembly of successive amino acids was performed under microwave radiation using O-(6-chlorobenzotriazol-1-yl)N,N,N',N'-tetramethyluronium hexafluorophosphate (HCTU) as coupling reagent and N,N-diisopropylethylamine (DIEA) in NMP as base. Fmoc deprotection was performed under microwave radiation with piperidine (20% v/v) in DMF. Finally, the beads were washed with dichloromethane (DCM) twice, methanol (MeOH) twice and DCM. After peptide assembly, resin beads were treated with 10 ml of a mixture of trifluoroacetic acid (TFA)/tri-isopropylsilane (TIS)/H_2_O 95/2.5/2.5 v/v/v, with the addition of 0.25 g of dithiothreitol (DTT) in the presence of cysteine, at room temperature for 3 h. The cleavage solution was concentrated and precipitated in 20 ml of cold diethyl ether. The crude product was filtered, washed with 10 ml of cold diethyl ether twice, dissolved in an H_2_O/acetonitrile (ACN) 95/5 v/v (0.1% TFA) mixture and freeze dried. Crude products were purified by preparative reverse-phase high performance liquid chromatography (RP-HPLC) on a Perkin Elmer LC200 system equipped with a 10 μm Ace Phenyl-300 column (HiChrom Ltd, Berkshire, UK) using a linear gradient of ACN in water containing 0.1% TFA. Purified peptides was characterized by matrix-assisted laser desorption ionization time-of-flight (MALDI-TOF) mass spectrometry at the Protein and Nucleic Chemistry Facility (University of Cambridge, Cambridge, UK).

### 2.2 Photoreactive triple helical peptides

#### 2.2.1 End-stapling of peptide strands

A mixture of Fmoc-GFGEEG-OH (4.5 mg, 5.6 × 10^−6^ mol), 1-[bis(dimethylamino)methylene]-1H-1,2,3-triazolo[4,5-b]pyridinium3-oxid hexa-fluorophosphate (HATU, 6.3 mg, 1.67 × 10^−5^ mol), 1-hydroxybenzotriazole (2.6 mg, 1.67 × 10^−5^ mol) and DIEA, (5.8 μl, 3.3 × 10^−5^ mol) was prepared in 5 ml of DMF. The mixture was added to resin beads bearing 5 × 10^−5^ mol of synthesised peptide and the reaction was left at room temperature for 24 h. The resin was filtered, a freshly prepared mixture added, and the reaction left a further 24 h. The operation was repeated once more, bringing the total reaction time to 3 days. Resin beads were washed with DCM twice, MeOH twice, then DCM. Removal of the Fmoc protective group was performed using 20% piperidine in DMF for 45 min. The resin was further washed with DCM twice, MeOH twice and DCM. Beads were then treated with 10 ml of a mixture of TFA/TIS/H_2_O, 95/2.5/2.5 v/v/v for 3 h. The cleavage solution was concentrated and precipitated in 20 ml of cold diethyl ether. The crude product was filtered, washed with 10 ml of cold diethyl ether, dissolved in an H_2_O/ACN 95/5 (0.1% TFA) mixture and freeze-dried. Crude products were purified by preparative RP-HPLC. The final products were characterized by HPLC and MALDI.

#### 2.2.2 Synthesis of photo-reactive triple helical peptides

The end-stapled triple-helical peptide (6.5 mg, 6.15 × 10^−7^ mol) was dissolved in 100 μl of dry DMF away from light. DIEA (0.2 μl, 1.23 × 10^−6^ mol) and NHS-Diazirine (0.3 mg, 1.23 × 10^−6^ mol, Life Technology) were added to the mixture. The reaction was left overnight at room temperature away from light. 1 ml of cold diethyl ether was added, resulting in the precipitation of a white solid. The mixture was filtered, the precipitate washed with 1 ml of cold diethyl ether, dissolved in H_2_O/ACN 95/5 and 0.1% TFA and freeze dried to yield 6.1 mg of white powder. The resulting product was further purified by dialysis in 0.01 M acetic acid (AcOH) during 4 h three times using a Float-A-Lyzer 500-1000 MWCO (Spectrum-Labs). After freeze-drying, 5.7 mg (5.4 × 10^−7^ mol) of the expected product was recovered as a white powder (87% yield).

### 2.3 Peptide melting temperature measurement by polarimetry

Peptides were solubilized in 900 μl of 10 mM phosphate buffer (with 150 mM NaCl) at a concentration of 2 mg/ml and the pH adjusted to 7.4. Peptide solutions were heated to 60 °C for 45 min to unfold the triple helix and kept at 4 °C overnight to refold. The melting temperature (T_m_) was measured by heating THP solutions from 8 °C to 80 °C at a ramp-rate of 1 °C/min in an Autopol III polarimeter. Optical rotation was measured every 15 s. T_m_ was determined by plotting the optical rotation and its first derivative against temperature [41].

### 2.4 Cell lines and culture conditions

Human fibrosarcoma cells, HT1080, were obtained from the European Collection of Animal Cell Cultures (Porton Down, UK). Rugli cells, derived from a rat glioma, were a kind gift from Dr. J. Gavrilovic, University of East Anglia, UK. Both cell lines were maintained at 37 °C in Dulbecco's modified Eagle's medium (DMEM, Sigma–Aldrich) containing 10% (v/v) foetal bovine serum (Sigma–Aldrich) and 1% (v/v) PenStrep (Life Technologies). Prior to cell binding or cell spreading experiments, cells were detached from cell culture flasks at a confluence of about 75% with 0.05% trypsin/0.02% EDTA (GE Healthcare), washed and resuspended in serum free DMEM.

### 2.5 Competition binding assay for α_2_ I-domain

The production and isolation of α_2_ I-domain as the glutathione S-transferase (GST) fusion protein has been described previously [42]. Immulon-2 HB 96-well plates (Thermo Scientific) were coated with 100 μl of GPC(GPP)_5_GFOGER(GPP)_5_GPC at 10 μg/ml in 0.01 M AcOH, or bovine serum albumin (BSA) overnight at 4 °C. Wells were blocked with 200 μl of 50 mg/ml BSA in tris-buffered saline (TBS; 50 mM Tris, 140 mM NaCl) for 1 h, then washed three times with adhesion buffer (TBS plus 1 mg/ml BSA and 5 mM MgCl_2_). The α_2_ I-domain GST was diluted to 0.5 μg/ml in adhesion buffer and incubated with 100 μg/ml of free peptide as indicated for 30 min at room temperature. 100 μl of the α_2_ I-domain GST/peptide mixture was then added to the GPC(GPP)_5_GFOGER(GPP)_5_GPC-coated wells for 1 h at room temperature. After washing three times with 200 μl of adhesion buffer, bound α_2_ I-domain GST was quantified by adding 200 μl of horseradish peroxidase-conjugated anti-GST antibody (Amersham Bioscience UK Ltd) diluted in adhesion buffer to 1:10,000 for 45 min at room temperature. Wells were washed four times with 200 μl of adhesion buffer and developed using 100 μl of TMB substrate (Thermo Scientific). The reaction was stopped with 100 μl of 2.5 M sulphuric acid and A_450_ was measured [43].

### 2.6 Collagen film preparation

#### 2.6.1 Film coating on 96-well plates

Collagen I from bovine achilles tendon (Sigma, #4387) was suspended at 0.5% w/v in 0.05 M AcOH and swollen overnight at 4 °C. The resulting suspension was homogenized using an Ultra-turrax VD125 blender at 13500 rpm for 20 min then centrifuged 5 min at 2500 rpm on a Hermle Z300 bench top centrifuge. It was further homogenized (10 min at 13500 rpm) and centrifuged again (5 min at 2500 rpm) to remove air bubbles. The resulting slurry was left overnight at room temperature before use. Immulon-2 HB 96-well plates were coated with 100 μl of slurry and were left to dry in a fume hood to yield 10 μm thick collagen films.

#### 2.6.2 Collagen film cross-linking

Cross-linking was carried out in 75% ethanol using a EDC/NHS/collagen molar ratio of 5/2/1 (1.15 g EDC and 0.276 g NHS per gram of collagen) as previously described [44], here termed 100% cross-linked, or 25/10/1 (5.75 g EDC and 1.38 g NHS per gram of collagen), termed 500% cross-linked films. The reaction was left at room temperature for 2 h and wells were washed twice with ethanol for 20 min, then three times with deionized water. Plates were then left to dry overnight in a fume hood.

### 2.7 Peptide-derivatized collagen films

Peptides in 5 mg/ml stock solution in 0.01 M AcOH were diluted to 5 μg/ml in PBS (pH 7.4) unless stated otherwise (for concentration studies, peptides were diluted to between 0.1 and 10 μg/ml as indicated). 100 μl of peptide solution was added to wells containing collagen films and incubated for 30 min in the dark at room temperature. Preliminary experiments indicated that incubation of peptide with a collagen scaffold for 30 min was optimal for passive absorption of a similar peptide (L. Mullen, PhD Thesis, University of Cambridge, 2010), and this incubation time was adopted for the experiments described here. Wells were then placed under a long-wavelength UV lamp (Blak-Ray B100AP, 365 nm wavelength) for 2–60 min, and routinely for 5 min, as indicated. Negative control wells were covered in aluminium foil to restrict UV exposure. Following UV treatment, wells were washed 3 times 2 min with 200 μl citrate buffer (pH 3) containing 1 mg/ml BSA and three times with 200 μl of PBS containing 1 mg/ml BSA. 100 μl of Streptavidin-Peroxidase Polymer Ultrasensitive (Sigma #S2438) diluted in PBS to 1:10000 were added to the wells and incubated 45 min at room temperature. After washing four times with 200 μl of PBS containing 1 mg/ml BSA, 100 μl of TMB substrate was added, the reaction stopped with 100 μl of 2.5 M sulphuric acid and A_450_ was measured.

### 2.8 α_2_ I-domain binding to functionalized collagen films

Peptide-derivatized collagen films were prepared as described above. 100 μl of the recombinant α_2_ I-domain-GST at 5 μg/ml, in TBS buffer containing either 5 mM Mg^2+^ or 5 mM EDTA, were added to wells and incubated for 90 min at room temperature. After washing three times with PBS containing 1 mg/ml BSA, 100 μl of the anti-GST HRP-conjugated antibody diluted 1:10,000 was added for 45 min at room temperature. After washing four times with 200 μl of PBS containing 1 mg/ml BSA, 100 μl of TMB substrate were added, the reaction was stopped with 100 μl of 2.5 M sulphuric acid and A_450_ was measured [43].

### 2.9 Cell binding to GFOGER-decorated collagen films

Peptide-decorated collagen films were prepared as described above. 100 μl of a suspension of Rugli or HT1080 cells at 5 × 10^5^ cells/ml in DMEM, containing either 5 mM Mg^2+^ or 5 mM EDTA, were added to wells. Cells were incubated at 37 °C for 20 min (HT1080) or 30 min (Rugli). Wells were twice washed gently with 200 μl of PBS. 150 μl of lysis buffer (containing 21 mg/ml disodium citrate, 6 mg/ml citric acid, 0.1% Triton X-100 and 5 mM *p*-nitrophenyl phosphate) were added for 90 min at room temperature. The reaction was terminated with 50 μl of 2 M sodium hydroxide and A_405_ was measured [43].

### 2.10 Cell spreading on collagen films

Collagen films were prepared on 13 mm diameter glass cover slips using 175 μl of collagen slurry, cross-linked, and functionalized with peptides as described above. 530 μl of Rugli or HT1080 cell suspension at 2 × 10^5^ cells/ml in DMEM, containing either 7.5 mM Mg^2+^ or 7.5 mM EDTA, were added. Cells were incubated at 37 °C until complete spreading was observed in control wells coated with the control THP, GPC(GPP)_5_GFOGER(GPP)_5_GPC, or until binding was observed on untreated collagen (around 45 min for HT1080, 90 min for Rugli cells). Cells were fixed by adding formaldehyde (37%) to a final concentration of 3% for 20 min and then washed three times with 200 μl of PBS. Cells were permeabilized by adding 400 μl of 0.5% Triton X-100 for 5 min, then stained with 400 μl of Rhodamine–Phalloidin (0.2 U/ml in PBS containing 0.1% BSA) during 45 min. Fluorescent images were obtained using an Olympus FV300 laser-scanning confocal microscope. For each condition, the area covered by cells in 10 randomly selected fields of view was measured using ImageJ software. The percentage area coverage and the average cell area were calculated as total cell area divided by the area of the field of view, and by the number of cells per field (counted manually), respectively. Phase contrast images were obtained using a Leica DMI6000 microscope.

### 2.11 Statistical analysis

Values shown are mean ± standard deviation, from up to nine separate experiments, each with three triplicate measurements. Mean values were compared using Prism software (GraphPad, San Diego) and 2-way ANOVA with Tukey's post-tests. Unless stated otherwise in Figure Legends, * denotes p < 0.05; ** denotes p < 0.01, *** denotes p < 0.001 and **** denotes p < 0.0001.

## 3 Results

### 3.1 Cell adhesion to EDC/NHS cross-linked collagen films

**Fig. 1:**
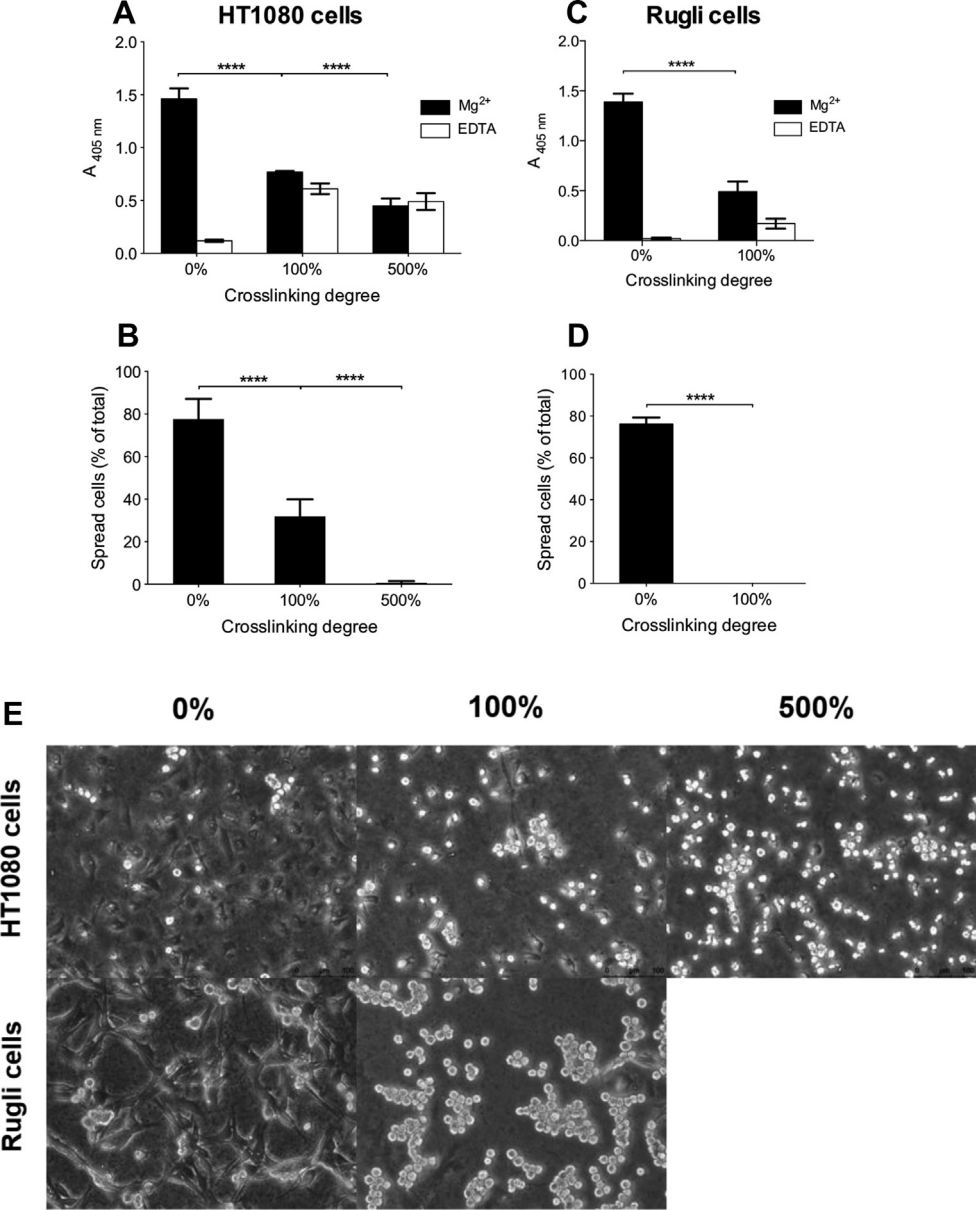
Binding and spreading of HT1080 (A, B) and Rugli cells (C, D) on collagen films. Cells were added to wells coated with collagen films, cross-linked (see Materials and methods) as indicated. For cell binding assays, 5 × 10^5^ cells/ml were added in the presence of 5 mM of Mg^2+^ or EDTA, during 20 min for HT1080 (A) or 30 min for Rugli (C) before lysis and quantification. For cell spreading assays, 2 × 10^5^ cells/ml were added in the presence of 7.5 mM Mg^2+^ or EDTA during 45 min for HT1080 (B) or 90 min for Rugli (D). Representative images of HT1080 and Rugli cells spread on cross-linked collagen films are shown in (E).

HT1080 cells attach to native collagen primarily through integrin α_2_β_1_ in a cation-dependent manner [45], [46]. Therefore HT1080 attachment to and spreading on EDC/NHS cross-linked collagen films was analysed in the presence and absence of Mg^2+^ as a measure of cellular integrin α_2_β_1_ engagement (Fig. 1A, B). Mg^2+^-dependent HT1080 attachment to EDC/NHS cross-linked collagen films was markedly diminished by pre-treatment of films with EDC to about half control values using 100%, and to one third using 500% cross-linking conditions (p < 0.0001, two-way ANOVA). This residual cell attachment to collagen after EDC/NHS cross-linking was Mg^2+^-independent, similar level being observed in the presence of EDTA, whilst binding to cross-linked collagen in the presence of EDTA was higher than to untreated collagen, a phenomenon under separate investigation. A similar, progressive effect on the spreading of HT1080 cells was observed following EDC/NHS cross-linking of collagen films. Cells adopted two morphologies: spread and extended cells that were phase-contrast dark, or spherical and refractile, that were light. The percentage of spread cells decreased from 78% in the absence of cross-linking to 32% and 0.7% with 100% and 500% EDC/NHS cross-linking respectively (p < 0.0001, two-way ANOVA). Representative images are shown in Fig. 1E.

To investigate the effect of EDC/NHS cross-linking on integrin α_1_β_1_ engagement with collagen we measured Rugli cell adhesion and spreading. Both parameters were more sensitive to EDC treatment than was observed with HT1080 cells, with cell attachment reduced to about one third of control levels, but spreading abolished, by 100% EDC/NHS cross-linking (Fig. 1C, D, p < 0.0001, two-way ANOVA) as well as 500% (data not shown). Representative images are shown in Fig. 1E.

It can be concluded that EDC/NHS cross-linking of collagen films inhibits integrin α_1_β_1_-and α_2_β_1_-dependent cell attachment and spreading.

### 3.2 Synthesis of UV-active THPs

#### 3.2.1 Triple helical peptide synthesis

**Fig. 2:**
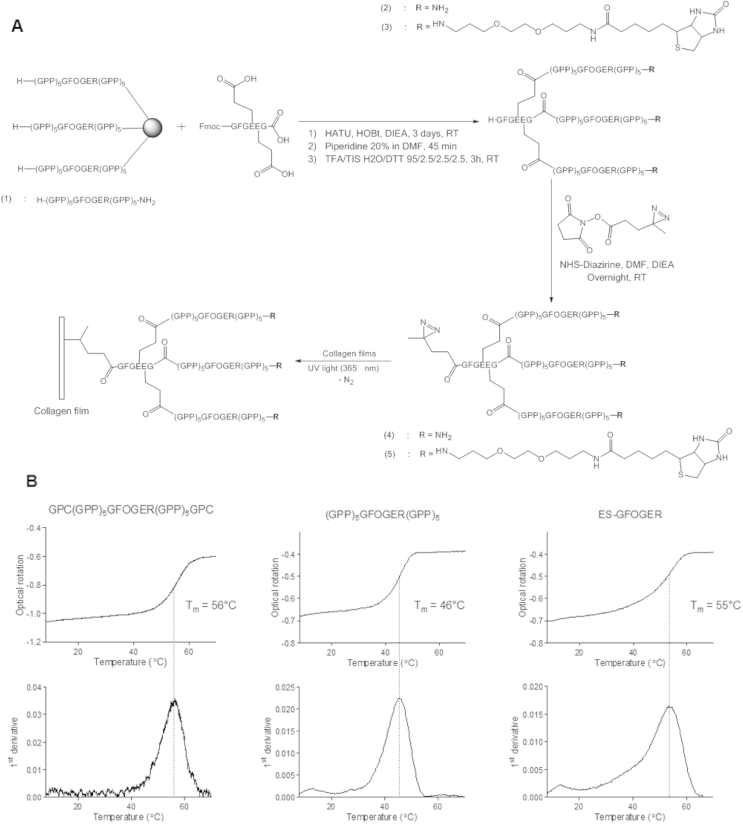
A) Synthetic pathway for the production of THPs and covalent linkage to collagen films. Compound (1): (GPP)_5_GFOGER(GPP)_5_; (2): ES-GFOGER; (3): ES-GFOGER-Biotin; (4): Diazirine-ES-GFOGER; (5): Diazirine-ES-GFOGER-Biotin. B) THP melting temperature (T_m_) measured by polarimetry, as described in Materials and Method. The upper row plots optical rotation (in degrees) versus temperature (°C) and the lower row shows the corresponding first derivative curves. T_m_ for GPC(GPP)_5_GFOGER(GPP)_5_GPC: 56 °C, for (GPP)_5_GFOGER(GPP)_5_: 46 °C, and for ES-GFOGER: 55 °C.

The protocol for the N-terminal end-stapling of peptide strands within a triple helix was adapted from Khew et al. [26], [27] and optimized in order to drive the reaction to completion (Fig. 2A). Best results were obtained using HATU-mediated coupling of the peptide tri-acid, Fmoc-GFGEEG, to the peptidyl resin over 3 days on Tantagel resin with a low loading (0.19 mmol g^−1^). The Fmoc protecting group was removed with piperidine in DMF, and the resin was cleaved using a TFA/TIS/H_2_O cocktail. The end-stapled (ES) THP was then recovered by evaporation of the TFA, precipitation in cold ether and freeze-drying. The crude product was finally purified by preparative HPLC.

For the purpose of this work, we synthesized several THPs containing the integrin-binding sequence, GFOGER [12], [47]. This motif was included in a host peptide containing five GPP triplets at both the C- and N-termini, giving the simplest THP used here, (GPP)_5_GFOGER(GPP)_5_, (compound 1, Fig. 2A). End-stapling of a similar peptide using Fmoc-GFGEEG led to the covalently-linked peptide, ES-GFOGER (compound 2, Fig. 2A). A related control peptide included additional GPC triplets, GPC(GPP)_5_GFOGER(GPP)_5_GPC; a peptide design we have used widely in the past, for example in Ref. [48]. Peptides containing a biotin group grafted to the C-terminus were also synthesized, enabling streptavidin-mediated detection on collagen films or scaffolds. A 20-atom linker, to prevent interaction between biotin and the active GFOGER motif was included. Once end-stapled, ES-GFOGER-Biotin (compound 3, Fig. 2A) was obtained.

#### 3.2.2 Triple helix stability

To characterize the effect of N-terminal end-stapling of a THP on its thermal stability, T_m_ was measured by polarimetry. Previous work has shown that a homogeneous population of triple helices ideally gives a sigmoidal optical rotation curve as the temperature rises [41]. T_m_ was compared for (GPP)_5_GFOGER(GPP)_5_ and its end-stapled equivalent, ES-GFOGER, and were found to be 46 °C for (GPP)_5_GFOGER(GPP)_5_ and 55 °C for ES-GFOGER, a difference of 9 °C resulting from end-stapling (Fig. 2B). T_m_ of the control GPC(GPP)_5_GFOGER(GPP)_5_GPC was measured as 56 °C, the difference between this and (GPP)_5_GFOGER(GPP)_5_ being due to inter-strand disulfide bridges, as previously described [49].

#### 3.2.3 Addition of Diazirine to end-stapled THPs

The photo-reactive group, Diazirine, was selected, being sensitive to long UV wavelength (330–370 nm), which minimizes the risk of damaging or altering the collagen substrate. It also has better stability in visible light than most photo-activatable groups but has a quicker activation time (half-life of 4 min at 8 W) [50]. Under UV light, Diazirine forms a highly-reactive carbene intermediate which readily adds to any nearby carbon skeleton, generating a covalent bond with just a short and simple residual link (Fig. 2A). Gaseous nitrogen is generated as a non-toxic product of the reaction.

Diazirine was grafted to the N-terminal primary amine of ES-GFOGER using the NHS-Diazirine precursor, added in a minimum volume of dry DMF in the presence of DIEA. An excess was used so that only NHS-Diazirine and the final compound Diazirine-ES-GFOGER remained in the mixture, which can easily be isolated by dialysis after precipitation in diethyl ether and freeze-drying (compound 4, Fig. 2A). An analog containing biotin on the C-terminal ends of the peptide was also produced (Diazirine-ES-GFOGER-biotin, compound 5, Fig. 2A).

### 3.3 Peptide binding to the α_2_ I-domain

The ability of the various derivatized THPs to bind to the integrin α_2_ I-domain, the active binding site of integrin α_2_β_1_, was tested in competition assays to ensure that the peptide–integrin interaction was not compromised by the various modifications descried above. The α_2_ I-domain was pre-incubated with THPs, then allowed to interact with the immobilized GPC(GPP)_5_GFOGER(GPP)_5_GPC, testing the ability of the end-stapled, Diazirine-derivatized and biotinylated peptides to bind the α2 I-domain in solution, and consequently inhibit its interaction with immobilised GFOGER.

**Fig. 3:**
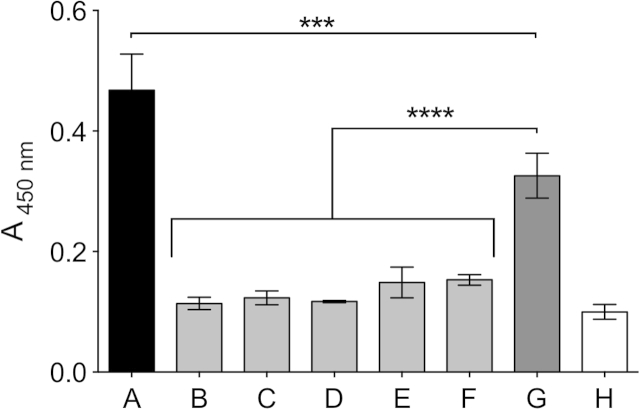
THP affinity for the α_2_ I-domain. The recombinant α_2_ I-domain-GST-fusion protein was incubated at 0.5 μg/ml with THP at 100 μg/ml for 30 min in the presence of Mg^2+^, with: no peptide (A); GPC(GPP)_5_GFOGER(GPP)_5_GPC (B); (GPP)_5_GFOGER(GPP)_5_ (C); ES-GFOGER (D); Diazirine-ES-GFOGER (E); Diazirine-ES-GFOGER-Biotin (F); or GPP10 (G). The adhesion of the α_2_ I-domain to immobilized GPC(GPP)_5_GFOGER(GPP)_5_GPC after 1 h was assessed using anti-GST antibody. Control wells were coated with BSA incubated with the α_2_ I-domain (H).

In the absence of dissolved peptides, α_2_ I-domain binding to coated GPC(GPP)_5_GFOGER(GPP)_5_GPC gave an absorbance of 0.47 ± 0.06. Pre-incubation of α_2_ I-domain with an inactive THP, GPC(GPP)_10_GPC, (GPP10), resulted in a modest reduction in binding to coated GPC(GPP)_5_GFOGER(GPP)_5_GPC an absorbance of 0.33 ± 0.04 (two way ANOVA, p < 0.001), which represents the true control level. However, incubation with the free GPC(GPP)_5_GFOGER(GPP)_5_GPC lowered the absorbance to 0.11 ± 0.01 (p < 0.0001, two-way ANOVA), close to the BSA negative control value. Likewise, incubation with other free peptides ((GPP)_5_GFOGER(GPP)_5_, ES-GFOGER, Diazirine-ES-GFOGER or Diazirine-ES-GFOGER-biotin) blocked α_2_ I-domain binding to the immobilized integrin-binding peptide to a similar level, giving absorbance between 0.12 and 0.15 (p < 0.0001, two-way ANOVA), showing that the observed inhibitory effect of the various peptides was specific to the α_2_ I-domain–GFOGER interaction, and suggesting that their affinities for the targeted integrin were broadly similar (Fig. 3).

### 3.4 Peptide functionalization on collagen films

Photo-reactive THPs were covalently coupled, by UV irradiation, to 100% EDC/NHS cross-linked collagen films, and C-terminal biotinylation of each peptide strand allowed them to be detected. Passive peptide adsorption was detected in the absence of UV treatment. An analog without Diazirine, ES-GFOGER-Biotin, was included as a negative control to assess non-specific UV-activated peptide–collagen binding. The experiment also included non-derivatized collagen films and BSA controls.

Washing steps were critical to remove loosely-bound, passively adsorbed peptides, so as to resolve covalently-linked THPs. Increasingly stringent washing conditions decreased the passive adsorption of peptides without UV irradiation or which lacked Diazirine. In contrast, the binding of UV cross-linked Diazirine-ES-GFOGER-Biotin was insensitive to increasing washing stringency (data not shown). An optimized procedure, consisting of three washes with citrate buffer containing BSA for 2 min, followed by three washes with PBS, was adopted in all further assays.

**Fig. 4:**
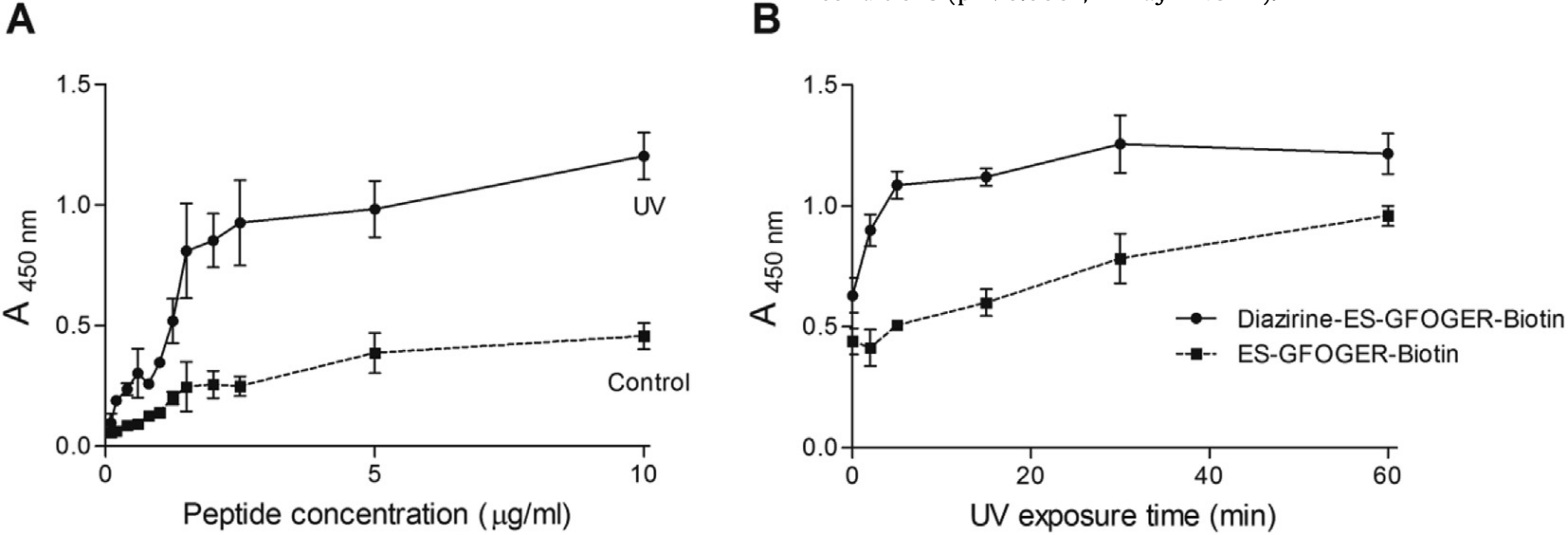
Optimization of conditions for UV binding of THPs to collagen films. Biotinylated THPs were added to wells coated with 100% EDC/NHS cross-linked collagen films. Following PBS and citrate buffer washings, attached peptides were detected through HRP-Streptavidin binding to Biotin. A) Diazirine-ES-GFOGER-Biotin was incubated at concentrations ranging from 0 to 10 μg/ml and exposed to long wavelength UV light for 5 min. B) Diazirine-ES-GFOGER-Biotin or ES-GFOGER-Biotin were added to collagen films at 5 μg/ml and exposed to long wavelength UV light for durations ranging from 0 to 60 min.

The amount of THP required for efficient derivatization of collagen films was determined using Diazirine-ES-GFOGER-Biotin, from 0 to 10 μg/ml, equivalent to 0 to 9.4 × 10^−7^ M. Peptide binding rose rapidly at up to 1.5 μg/ml. Beyond this concentration, a plateau was reached at 2.5 μg/ml with an absorbance of 0.93 ± 0.18 (Fig. 4A). No further significant increase in binding was observed, tested to 10 μg/ml. For all concentrations, higher peptide retention on cross-linked collagen films was observed after UV treatment.

The UV treatment time for Diazirine-ES-GFOGER-Biotin (5 μg/ml) retention on films was optimised, using the non-photo-reactive ES-GFOGER-Biotin as a negative control (Fig. 4B). A clear increase in Diazirine-ES-GFOGER-Biotin retention was observed for up to 5 min UV treatment, with only slight effect of any further increase in UV exposure. The retention of ES-GFOGER-Biotin was lower than of the Diazirine-containing peptide, but increased linearly with extended irradiation time, up to 60 min. Therefore, to optimize selective Diazirine-mediated peptide binding to collagen films, a UV exposure time of 5 min was chosen.

**Fig. 5:**
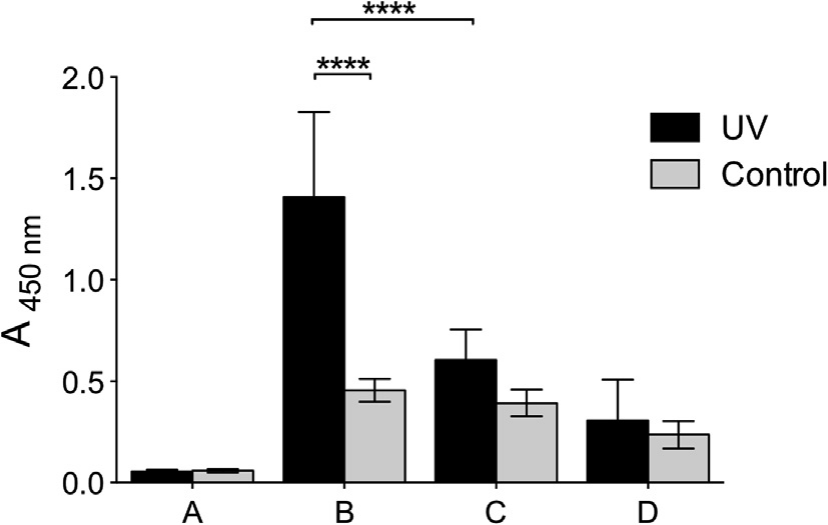
THP coating on collagen films using UV treatment. Biotinylated THPs were added at 5 μg/ml to wells coated with 100% EDC/NHS cross-linked collagen films and exposed to UV light for 5 min. Following PBS and citrate buffer washings, attached peptides were detected through HRP-Streptavidin binding to Biotin. Pooled means ± SD were calculated from three repeat experiments. Wells were coated with collagen films without peptides (A); in the presence of Diazirine-ES-GFOGER-Biotin (B); or in the presence of ES-GFOGER-Biotin (C). Control wells were coated with BSA in the presence Diazirine-ES-GFOGER-Biotin (D). Diazirine-ES-GFOGER-Biotin followed by UV treatment led to a significant increase in peptide retention compared to all other conditions (p < 0.0001, 2-way ANOVA).

Using previously defined conditions, a clear increase in peptide retention was observed for peptides bearing Diazirine after UV irradiation (Fig. 5). In six separate experiments, the absorbance of Diazirine-ES-GFOGER-Biotin reached 1.41 ± 0.42, a value three times higher than without UV treatment (0.46 ± 0.11, p < 0.0001, 2-way ANOVA). Without UV treatment, the retention of Diazirine-ES-GFOGER-Biotin was similar to that of the non-photo-reactive peptide ES-GFOGER-Biotin with or without UV treatment (0.61 ± 0.15 and 0.39 ± 0.13 respectively). These values indicate passive adsorption of peptides in films incubated with peptide that lacked Diazirine or were not exposed to UV light, by comparison with non-derivatized collagen films (0.06 ± 0.01).

### 3.5 Binding of the α_2_ I-domain to peptide derivatized films

**Fig. 6:**
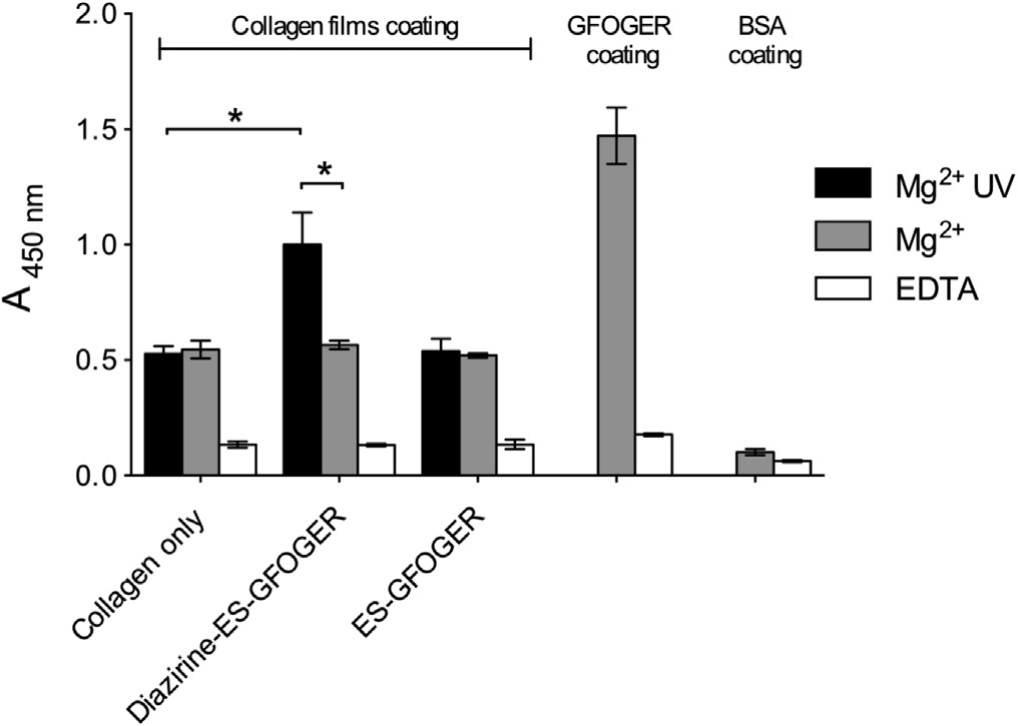
α_2_ I-domain binding to peptide derivatized collagen films. Wells were coated with 100% EDC/NHS cross-linked collagen films. Diazirine-ES-GFOGER or ES-GFOGER were added to films at 5 μg/ml and exposed to UV light for 5 min. Controls include wells coated with GPC(GPP)_5_GFOGER(GPP)_5_GPC or BSA. I-domain-GST fusions were incubated on collagen films at 5 μg/ml for 90 min in the presence of 5 mM Mg^2+^ or EDTA and detected using an anti-GST antibody. A representative dataset from three identical experiments is shown (n = 3 for each condition).

The binding of α_2_ I-domain was evaluated on 100% EDC/NHS cross-linked collagen films functionalized with THPs as described above. To resolve the Mg^2+^-dependence of α_2_ I-domain binding, EDTA was included as a control, alongside BSA and GPC(GPP)_5_GFOGER(GPP)_5_GPC-coated control wells (Fig. 6). EDTA reduced α_2_ I-domain binding to near-background levels in all conditions tested. Crucially, UV irradiation of collagen films functionalized with Diazirine-ES-GFOGER doubled the Mg^2+^-dependent binding of the α_2_ I-domain (A_450_ = 0.94 ± 0.36) compared to all other conditions (A_450_ = ∼0.5) in three repeated experiments (p < 0.1, 2-way ANOVA). α_2_ I-domain binding to the control GFOGER coating was higher (1.14 ± 0.18) and was reduced to about 10% of this level by EDTA, close to background.

### 3.6 Cell behaviour on collagen films derivatized with THPs

#### 3.6.1 Cell binding assays

**Fig. 7:**
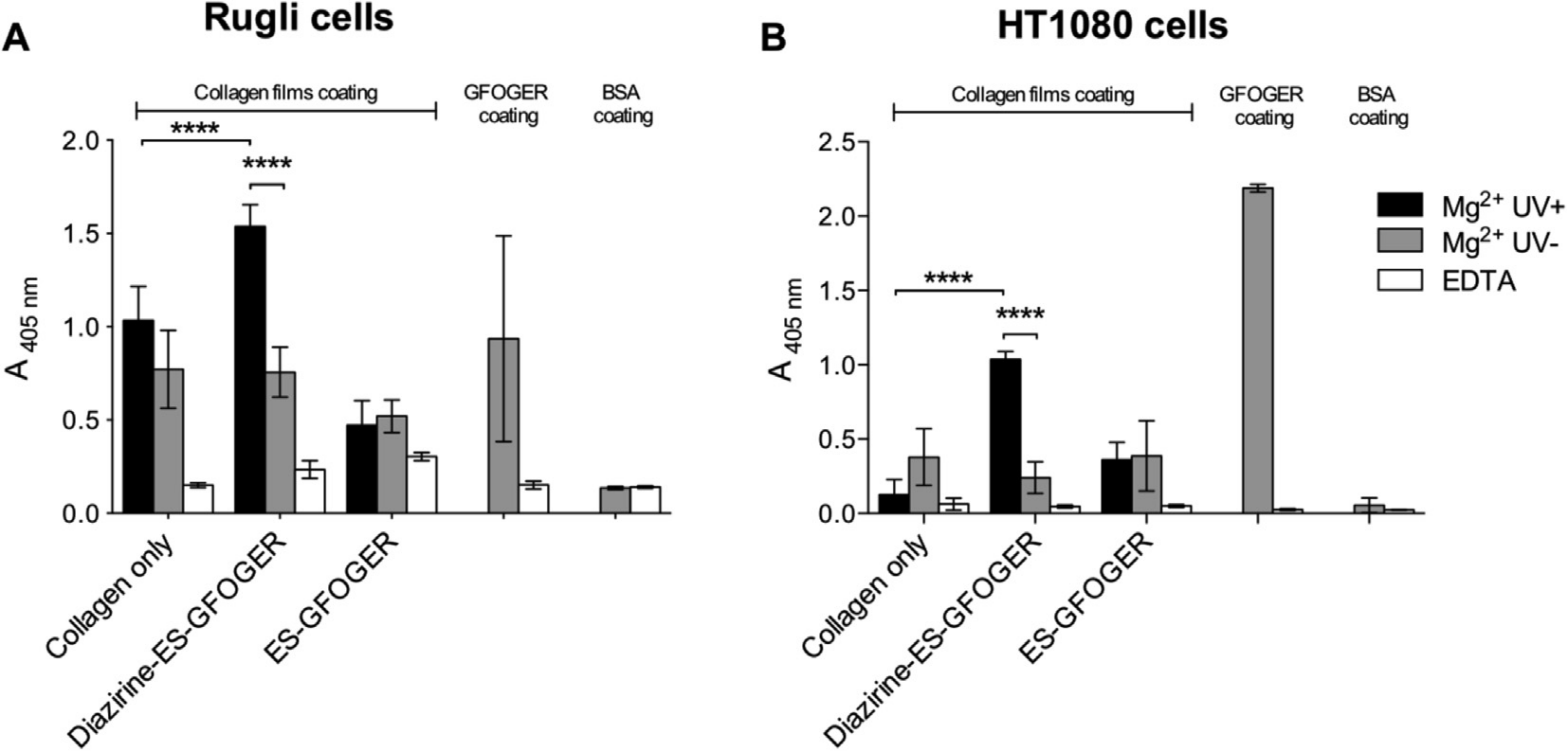
HT1080 and Rugli cell binding to peptide derivatized cross-linked collagen films. Diazirine-ES-GFOGER or ES-GFOGER were added to EDC/NHS cross-linked collagen films at 5 μg/ml and exposed to UV light for 5 min. Controls include wells coated with GPC(GPP)_5_GFOGER(GPP)_5_GPC or BSA. 5 × 10^5^ cells/ml were added to wells in the presence of 5 mM Mg^2+^ or EDTA, lysed and quantitated. A) Rugli cells binding to peptide derivatized 100% EDC/NHS cross-linked collagen films after 30 min; a representative dataset from seven independent experiments is shown (each in triplicate). B) HT1080 cells binding to peptide derivatized 500% EDC/NHS cross-linked collagen films after 20 min; a representative figure from nine independent experiments is shown (each in triplicate).

For all cell assays, collagen films were optimally derivatized with photoreactive THPs as described above. Rugli and HT1080 cell lines were used to examine the ability of attached peptides to ligate collagen-specific integrins, in the presence of Mg^2+^ (integrin-binding) or EDTA (non-specific attachment), with GPC(GPP)_5_GFOGER(GPP)_5_GPC-coated wells acting as positive controls. Initially, 100% EDC/NHS cross-linked collagen films were used (Fig. 7).

Similar levels of Rugli cell binding occurred on control collagen films and collagen films functionalized with ES-GFOGER or with Diazirine-ES-GFOGER without UV treatment, (A_405_ between 0.73 ± 0.33 and 1.02 ± 0.38, respectively, from 7 replicate experiments). In contrast, UV exposure of films functionalized with Diazirine-ES-GFOGER led to a significant, 1.7-fold increase in cell binding, with A_405_ reaching 1.55 ± 0.48 (p < 0.01, two-way ANOVA) compared with all other conditions. A representative experiment is shown in Fig. 7A.

The use of 100% EDC/NHS cross-linking did not completely ablate HT1080 cell binding to collagen films (Fig. 1). Therefore, we produced modified collagen films, employing 500% EDC/NHS cross-linking, as used for Fig. 1. HT1080 binding to these films was dramatically reduced compared to non cross-linked and 100% cross-linked films. Use of such films functionalized with Diazirine-ES-GFOGER under UV irradiation resulted in a 1.6-fold increase in HT1080 attachment compared to other treatments (p < 0.05, 2-way ANOVA, 9 replicated experiments). This was similar to the response observed for Rugli cells on 100% cross-linked films. A representative experiment is shown in Fig. 7B. Furthermore, pre-incubation with an α_2_β_1_ blocking antibody, 6F1, reduced the HT1080 binding to EDTA level, regardless of the presence of THPs (Supplementary Figure). This result confirms that HT1080 binding to the collagen films is integrin α_2_β_1_-mediated, and the increase observed in the presence of covalently linked THPs is due to the GFOGER-containing THP interacting with the targeted receptor, α_2_β_1_.

#### 3.6.2 Cell spreading assays

**Fig. 8:**
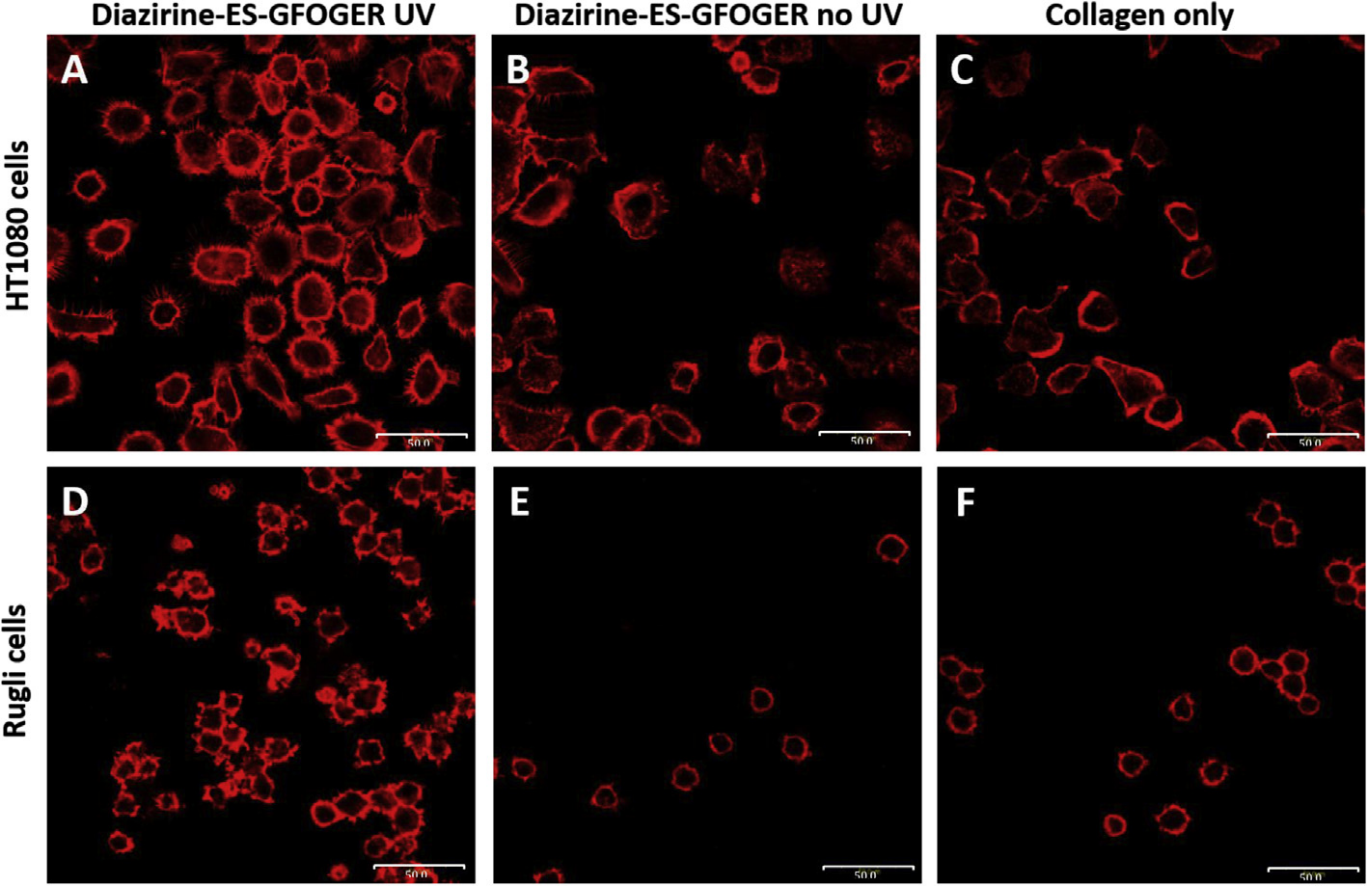
Confocal images of HT1080 and Rugli cell spreading on peptide derivatized collagen films. Diazirine-ES-GFOGER was added to EDC/NHS cross-linked collagen films at 5 μg/ml and exposed to UV light for 5 min. 2 × 10^5^ cells/ml are added to films in the presence of 7.5 mM Mg^2+^. (A–C): HT1080 cells after 45 min on 500% cross-linked films functionalized with Diazirine-ES-GFOGER following UV treatment, (A); and without UV treatment, (B); or on films without peptide (C). (D–F): Rugli cells after 90 min on 100% cross-linked films functionalized with Diazirine-ES-GFOGER following UV treatment (D) and without UV treatment (E), or on films without peptide (F). Representative fields of view from a single experiment are shown.

We show in Fig. 1 that both HT1080 and Rugli cells were able to bind to native collagen films, and that EDC/NHS cross-linking impairs cell spreading. We investigated whether covalent attachment of peptides could provide the relevant cues to restore cell spreading on such films. The percentage coverage of the field of view by cells was measured on EDC/NHS cross-linked collagen and collagen derivatized with Diazirine-ES-GFOGER, with or without UV treatment (Fig. 8). Cells were visualized by selectively staining actin with Rhodamine–Phalloidin, as described in Methods.

HT1080 spreading experiments were conducted on 500% EDC/NHS cross-linked collagen films, as described above. A difference in shape was observed between cells incubated on peptide-functionalized and control collagen films. In the presence of Mg^2+^, peptide-derivatized, UV-treated films supported spreading of the majority of cells, which displayed many filopodia extending from the cell body. In contrast, more HT1080 cells on control collagen films remained regular in outline, as did cells in the presence of EDTA.

**Fig. 9:**
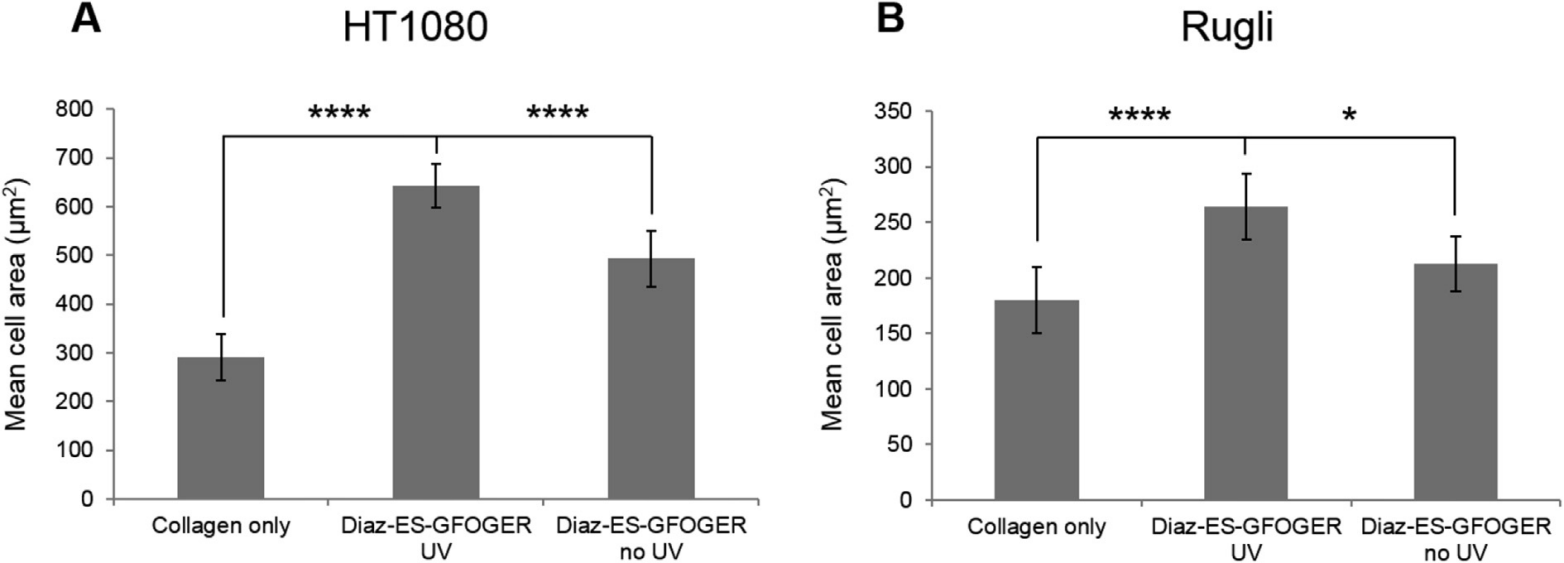
HT1080 and Rugli mean cell area (calculated from 10 fields of view for each conditions). Diazirine-ES-GFOGER are added to EDC/NHS cross-linked collagen films at 5 μg/ml and exposed to UV light for 5 min. 2 × 10^5^ cells/ml are added to films in the presence of 7.5 mM Mg^2+^. (A): Mean HT1080 surface area after 45 min on 500% cross-linked films. (B): Rugli cells after 90 min on 100% cross-linked films. In both cases, functionalization with Diazirine-ES-GFOGER led to a significant increase in cell surface area. Data are from the experiment shown in Fig. 8.

Cell surface coverage increased from 6% in the absence of peptide to 38% on peptide-derivatized, UV-treated films, demonstrating that covalently-bound peptides promote cell interaction with the substrate. Cell spreading occurred to a lesser extent on films incubated with photo-reactive peptides but not UV irradiated (15% surface coverage), indicating that some passive peptide adsorption to films could still occur which might support a degree of change in cell morphology. Likewise, the mean cell area, (calculated as total area of field covered by cells/cell number), increased 2.2-fold on UV- and Diazirine-ES-GFOGER-treated 500% cross-linked collagen films (p < 0.0001, two-way ANOVA). The average cell size was 291 μm^2^ in the absence of peptide, 643 μm^2^ on films coated with Diazirine-ES-GFOGER under UV and 493 μm^2^ for films unexposed to UV (Fig. 9A).

Rugli cell spreading was examined similarly, using 100% EDC/NHS cross-linked films. A clear difference in cell attachment and morphology (larger cells and more filopodia) was apparent between collagen films functionalized with UV-linked peptides (16% surface coverage) and unmodified films (1% surface coverage). Samples not subjected to UV exposure did not support much cell attachment (4% surface coverage) or spreading of those cells that did attach. The average cell size on 100% cross-linked collagen films also increased significantly in the presence of UV treated Diazirine-ES-GFOGER (264 μm^2^) compared to films without peptides (180 μm^2^, p < 0.0001, two-way ANOVA) or with Diazirine-ES-GFOGER unexposed to UV (213 μm^2^, p < 0.1, two-way ANOVA) (Fig. 9B).

## 4 Discussion

We confirm here that EDC/NHS treatment of collagen-based materials restricts the binding of HT1080 and Rugli cells, in line with published work [11], [12]. Our purpose was to develop a method to restore integrin-reactivity to such EDC/NHS treated collagen films by incorporating photo-activatable triple-helical peptide ligands that can be irreversibly covalently linked to collagen. This would be of direct benefit to the application of collagen scaffolds, and would provide proof of concept for enhancing the cell-reactivity of other inert biomaterials.

The first step was to synthesize these integrin ligands as end-stapled THPs. We adapted a synthetic pathway developed by Khew et al. [27], which involves the coupling of a tri-acid hexapeptide, Fmoc-GFGEEG, to three resin-supported (GPP)_5_GFOGER(GPP)_5_ peptide strands via their free N-termini. One advantage of such end-stapling is that it stabilizes the triple-helical conformation of the corresponding THP [27]. This effect is reflected by a 9 °C increase in melting temperature compared with (GPP)_5_GFOGER(GPP)_5_.

The synthetic strategy employed here provides a single reactive free amine at the N-terminus of a THP, to which other species of interest can subsequently be grafted. In this case, a photoreactive group, Diazirine, was used to allow subsequent covalent linkage to a collagen film. Unlike most other peptide cross-linking strategies, this allows the selective addition of one UV-reactive moiety to a single site on a triple helix. This leads to the very specific, directed incorporation of a pre-assembled and functional triple helix into a collagen structure. Since Diazirine can couple to any other carbon skeleton under suitable irradiation conditions, the same benefit would apply to the photochemical attachment of such THPs to other types of substrate, such as synthetic polymers that are also widely used in tissue engineering [51]. Similarly, single-point, one-to-one stoichiometric attachment to the GFGEEG tri-peptide might be exploited for the derivatization of ES-THPs with other molecules of interest, such as fluorophores.

Before functionalization of collagen films with THPs, we verified that the chemical modifications described above did not affect the binding to α_2_ I-domain. The different end-stapled THPs, ES-GFOGER and Diazirine-ES-GFOGER were each able to displace the receptor from immobilized GPC(GPP)_5_GFOGER(GPP)_5_GPC. Similarly, the presence of biotin on the C-termini of the peptide chains did not affect their α_2_ I-domain binding activity. This showed that our synthetic strategy yields integrin α_2_-reactive THPs that are suitable for further study.

We allowed photo-activatable THPs to adsorb passively onto collagen films [28], prior to UV treatment, to ensure close proximity between the peptide and the substrate, thus making the coupling reaction highly efficient. Covalent attachment will minimize subsequent elution into the medium and loss of integrin reactivity. It is possible that cross-linking of THPs to collagen may not be entirely Diazirine-mediated, but might also occur through UV-induced side reactions with aromatic residues in either peptide or collagen. The three aromatic residues, Tyr, Trp and Phe, are especially sensitive to short wavelength UV light, between 200 and 280 nm, generating highly reactive radicals enabling various photochemical side-reactions [52], [53]. Phe can dissociate to form a highly reactive benzyl radical, and is most relevant, since Tyr and Trp, although absorbing more strongly, occur far less frequently in the helical domains of the fibrillar collagens. It would be of concern if the phenylalanine side chain in the GFOGER motif was involved in such photoreactions [54], as integrin-reactivity would be compromised directly. Indeed, increasing covalent linkage between collagen and ES-GFOGER could be observed after extended exposure, despite using much longer wavelength, 365 nm, than the 200–254 nm absorption peak of the aromatic sidechains described above. Moreover, and beyond the scope of the present work, several binding sites for other collagen-binding proteins contain Phe residues, and are likely to be compromised by excessive UV irradiation. The shared (and major) site for the discoidin domain receptors (DDRs), for von Willebrand factor (VWF) and SPARC contains a critical GVMGFO motif [18] and the immune receptor OSCAR binds to a consensus motif, GPOGPAGFO [55].

To restrict such non-specific UV-induced effects, while allowing for maximal THP binding to collagen, the UV treatment time was optimized, and fixed at 5 min for further experiments. This optimal exposure to UV light caused only slight binding of ES-GFOGER-Biotin to collagen films, as significant peptide retention required both UV treatment and the presence of Diazirine. Maximal peptide derivatization was achieved at a concentration of 2.5 μg/ml, beyond which the films became saturated and a plateau was reached. In summary, our method converged upon 5 min exposure to UV and a THP concentration of 5 μg/ml.

Films functionalized with THPs following this protocol were incubated with the recombinant protein of the α_2_ I-domain, as the first step to investigate integrin reactivity on EDC/NHS cross-linked collagen substrate. Only the combination of Diazirine containing peptide and UV exposure led to an increase in the binding of α_2_ I-domain.

We examined the ability of these THPs to support binding of cells expressing specific collagen-binding integrins, Rugli cells (for α_1_β_1_) and HT1080 cells (for α_2_β_1_), which have been widely used for this purpose. Only the use of peptides bearing Diazirine and subsequent exposure to UV light restored Mg^2+^-dependent Rugli cell attachment to 100% EDC/NHS cross-linked films. With HT1080, however, residual cell binding occurred regardless of the presence of peptides.

Previous work here [56] indicated that 100% EDC/NHS cross-linking of collagen consumes about half of the 103 free amine (lysine residues) contained in each tropocollagen molecule. We assume, therefore, that 80 or more of its 138 glutamate residues remain free, even if glutamate was preferentially cross-linked over the 84 aspartate residues also present. Our binding data suggest that 100% cross-linking conditions leave sufficient intact integrin binding site to support some α_2_β_1_-mediated attachment. In contrast, 500% EDC/NHS cross-linked collagen supported very little cation-dependent cell adhesion, unless derivatized using Diazirine-ES-GFOGER and UV light.

The question arises as to whether derivatizaton with integrin ligand allows cells to re-establish functional contact with cross-linked films. We show first that the cross-linking process impairs cell interaction with collagen, since, although cells can attach, the majority remain spherical, do not spread, and are seen as refractile in Fig. 1. Hence, the cell-reactive collagen substrate has been degraded and become inert. Next, the derivatization of such collagen films with photo-reactive integrin ligand leads to increased cell attachment, shown for HT1080 cells to be α_2_β_1_-dependent using blocking antibodies. This does not necessarily indicate functional contact.

However, cell spreading has long been recognised as an active process [57], and requires reorganisation and extension of the cytoskeleton, a process that involves multiple small GTPases and protein kinase-mediated signals from the surrounding ECM, the recruitment of adaptor proteins, and culminates in the polymerization of soluble actin and the extension of filopodia and lamellipodia, reviewed by Price et al. [58], Yamaguchi and Condeelis [59] and Lawson and Burridge [60].

To address the capacity of derivatization to restore active cell spreading on cross-linked collagen films, we measured the area of attached cells microscopically, using Rhodamine/Phalloidin-stained Rugli and HT1080 cells, on films cross-linked to 100% and 500%, respectively, with or without Diazirine-ES-GFOGER/UV treatment. Photo-activated THP decoration of films resulted in a dramatic increase in cell area of both HT1080 and Rugli cells, not observed on collagen-only controls. On such films, both cell types displayed prominent filopodial extensions, with occasional actin filaments visible within the cytoplasm. Intermediate cell areas were observed on films to which Diazirine-ES-GFOGER was passively adsorbed without UV treatment. This confirms that covalent linkage of integrin-binding peptides can restore cell attachment and spreading on heavily cross-linked films, leading to the observed change in cell morphology. Overall, these cell spreading results are fully aligned with our earlier adhesion experiments, in which UV-linked THPs enhanced both α_2_ I-domain binding and cell binding through both α_1_β_1_ and α_2_β_1_.

We considered whether photochemical derivatisation might affect the ability of cells to interact with a collagen film in respects other than its integrin reactivity, perhaps by modifying its mechanical properties [10], [56]. We believe that very little mass will be incorporated into the film by derivatisation from low-concentration peptide solutions, so that its bulk density, porosity and compressive stiffness is likely to remain unchanged. The THP inserted is also charge-neutral. The design of our peptide with a single reactive Diazirine group per THP rather than one per strand (which would have been much simpler to synthesize) avoids the possibility of peptide-mediated photochemical covalent linkage between adjacent collagen strands, which could increase film stiffness much like the conventional EDC treatment. Despite these considerations, the mechanical properties of our materials are currently under test to verify these assumptions.

A second area of uncertainty is the mechanism by which cells interact with and spread upon our derivatized films. Our intention here was to use short-term adhesion experiments that would minimise re-synthesis of matrix by the cells under test, and simply to show restored integrin-reactivity. A longer-term and more comprehensive understanding of the different cellular adhesive axes will be necessary before we can fully conclude that normal, physiological processes are restored for each target cell type by coupling GFOGER to the biomaterial substrate. Davidenko et al. found, using platelets as a model system, that full adhesion was maintained if the EDC treatment of collagen scaffolds was simply reduced to 10%, whereas for HT1080 cells, such reduced EDC treatment caused a 30% loss of integrin-mediated cellular adhesion [61]. Here, we show on collagen films that Rugli cells are even more sensitive than HT1080s to EDC crosslinking. These differences may reflect both the presence of other adhesive processes and different levels of integrin expression in the cells under study. Platelet adhesion, reviewed by Nieswandt and Watson [62] is multifactorial, and known to include both α2β1 and GPVI, another collagen receptor that recognises GPO triplets. These are not subject to modification by EDC, and the adhesive process also requires VWF, discussed above, in some high-shear physiological settings. Platelet adhesion to collagen can be recapitulated by a surface coated with a collagen peptide mixture that binds these three axes [28]. Other collagen receptors, for example the DDRs, may be present on other cells, and are known to up-regulate integrin affinity [63]. Together, such cooperative processes may compensate for loss or partial loss of integrin reactivity.

Other plasma or matrix molecules may also contribute to cellular adhesion indirectly, bridging between collagen and the cell surface. VWF and fibronectin provide obvious examples, relevant to both platelets and the HT1080 cells used here. The sensitivity to EDC of their binding sites in collagen remains to be established. However, the VWF and DDR binding motif lies in residues 396 to 404, adjacent to a derivatizable lysine residue (K408 in the collagen helix), whilst the fibronectin binding site contains a glutamate (E791). Therefore, ablation of VWF, DDR or fibronectin binding to collagen by EDC treatment is a real possibility.

It should also be noted that the Rugli and HT1080 model cells, frequently used in the field because they express respectively α_1_β_1_ and α_2_β_1_, are both tumour cell lines that may be more inclined to collagen-responsive, invasive behaviour that includes the expression of collagen-degrading enzymes. These cell lines may over-represent the general cellular interaction with collagen.

It is important therefore, that the design of films and scaffolds, whether of collagen or other biomaterials, considers first the desired physical properties, then the adhesion molecules and processes expressed by the target cell type. The present work offers a means conferring collagen-binding integrin reactivity upon an inert material. The use of collagen motifs for other classes of receptor, for example GPVI or DDRs, will allow the methodology to be broadened, even to the inclusion of motifs that will recruit other cell-reactive matrix molecules. Finally, it should be emphasised that the construction of such biomaterials and their subsequent derivatization will need to be tailored to each particular cell type and application.

## 5 Conclusion

The goal of this study was to establish a methodology to restore cell activity to inert substrates, exemplified here by collagen films in which the native collagen–cell interaction had been ablated by carbodiimide cross-linking. We have developed a protocol for the synthesis and photochemical coupling of THPs onto a stable fibrous collagen I structure. These grafted peptides retained their affinity for the target receptors, the collagen-binding integrins. The behaviour of Rugli and HT1080 cells, expressing, respectively, integrins α_1_β_1_ and α_2_β_1_, showed that the presence of covalently-linked peptides enhanced cell binding and active cell spreading on such collagen films. The use of the GFOGER motif grafted to various types of scaffolds is applicable in principle to any kind of cells that express the integrins, α_1_-, α_2_-, α_10_-and α_11_-β_1_. Chondrocytes, for example, key targets in tissue engineering, express α_10_β_1_. In the present work, we have shown two possible examples using HT1080 and Rugli cells. Our method can readily be extended to cell lines expressing other receptors by grafting other photoactivatable ligands, able to promote cell growth and differentiation (e.g. with ligands targeting DDR1 and 2 as well as VWF or other ECM components). This work establishes a generic technology, not restricted to the collagen films, that can be used to link authentic collagen-derived synthetic peptides to substrates of interest. We anticipate that the method might readily be applied to two- and three-dimensional scaffolds, for diverse applications in cell culture, tissue engineering and regenerative medicine.
