# Supplementary material for: The synthesis and coupling of photoreactive collagen-based peptides to restore integrin reactivity to an inert substrate, chemically-crosslinked collagen
Source: Biomaterials. 2016 Apr;85:65–77. doi: 10.1016/j.biomaterials.2016.01.044 (PMC4773407; doi:10.1016/j.biomaterials.2016.01.044)
Supplement: Supplementary file 1 [file mmc1.docx]

**Supplementary data.**


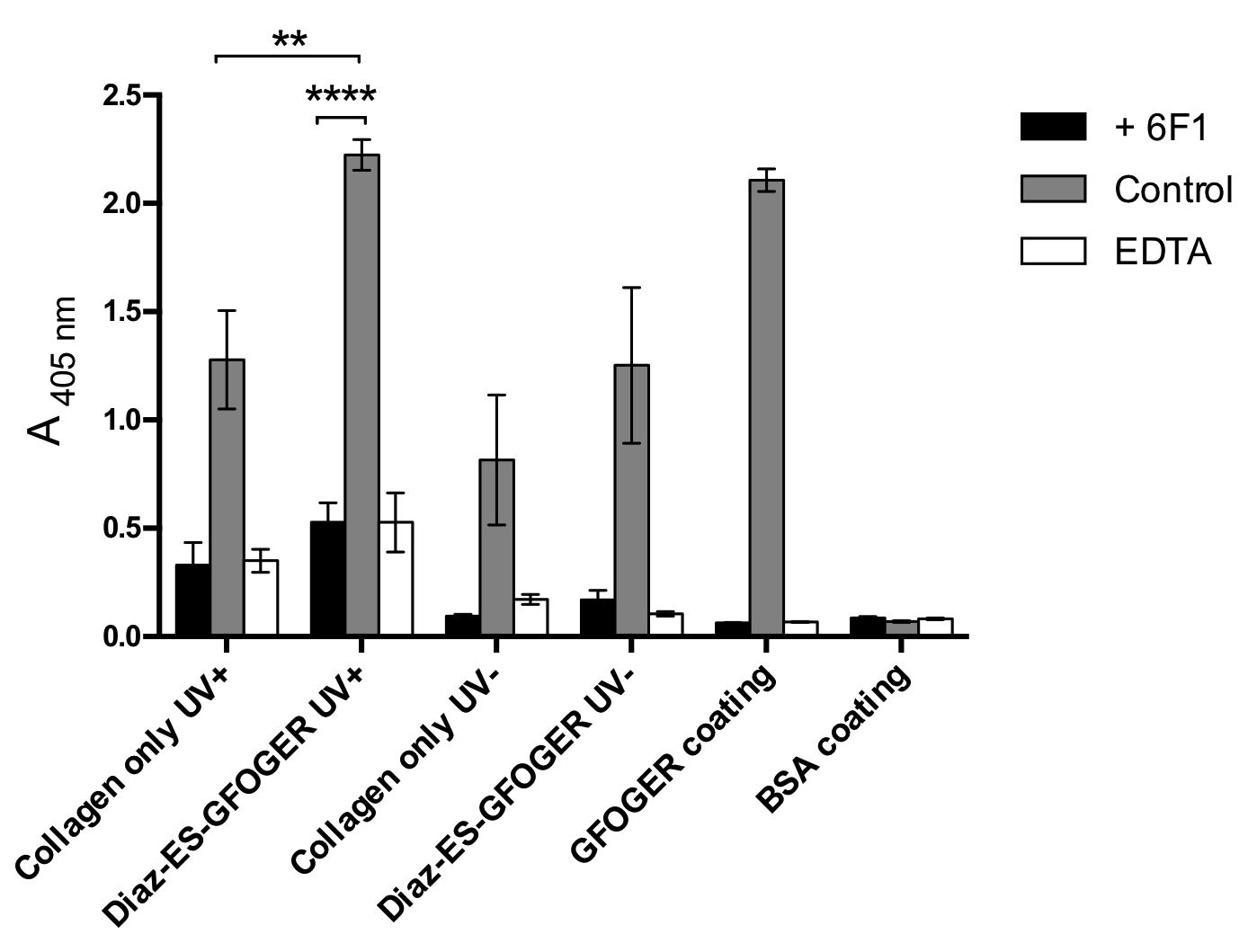


HT1080 cell binding to peptide derivatized cross-linked collagen films in the presence of 6F1, the α_2_β_1_*-*blocking antibody (the gift of Dr B Coller, NY). Diazirine-ES-GFOGER was added to 500% EDC/NHS cross-linked collagen films at 5 µg/ml and exposed to UV light for 5 min. Controls include wells coated with GPC(GPP)_5_GFOGER(GPP)_5_GPC or BSA. 5 x 10^5^ cells/ml were incubated for 20 min with 5mM Mg^2+^, 5 µg/ml 6F1 and 5 mM Mg^2+^ or EDTA. Cells were then added to wells, left for 20 min, lysed and quantitated. A representative dataset from three independent experiments is shown (n = 3 per repeat). Two-way ANOVA showed significant increase in the presence of Mg^2+^ without 6F1 compared to Mg^2+^ with 6F1 or EDTA (p < 0.001 for all conditions except for BSA control). Moreover, the presence of covalently linked Diaz-ES-GFOGER led to a further 1.5-fold increase in cell binding (p < 0.001 in two-way ANOVA), which is consistent with results showed in Figure 7B.
